# Time of Death Revealed by Hydrocarbons of Empty Puparia of *Chrysomya megacephala* (Fabricius) (Diptera: Calliphoridae): A Field Experiment

**DOI:** 10.1371/journal.pone.0073043

**Published:** 2013-09-05

**Authors:** Guang-Hui Zhu, Xiao-Jun Yu, Liang-Xing Xie, Hao Luo, Dian Wang, Jun-Yao Lv, Xiao-Hu Xu

**Affiliations:** Department of Forensic Medicine, Shantou University Medical College, Shantou, Guangdong, China; CNRS, France

## Abstract

Determination of the postmortem interval (PMI) is crucial for investigating homicide. However, there are currently *only limited methods* available. Especially, once the PMI exceeds the duration of pre-adult development of the flies with the adult emergence, its determination is very approximate. Herein, we report the regular changes in hydrocarbon composition during the weathering process of the puparia in the field in *Chrysomya megacephala* (Fabricius) (Diptera: Calliphoridae), one of the common species of necrophagous flies. Correlation analysis showed that the relative abundance of nearly all of the branched alkanes and alkenes decreased significantly with the weathering time. Especially, for 9 of the peaks, over 88% of the variance in their abundance was explained by weathering time. Further analysis indicated that the regular changes caused mainly by the different weathering rates of various hydrocarbons. Additionally, the weathering rates were found to depend on the chemical structure and molecular weight of the hydrocarbons. These results indicate strongly that hydrocarbon analysis is a powerful tool for determining the weathering time of the necrophagous fly puparia, and is expected to markedly improve the determination of the late PMI.

## Introduction

Estimation of the postmortem interval (PMI) is one of the most important tasks in death investigation. In a homicide case, it can help to identify the murderer, eliminate suspects and confirm an alibi [1], [2]. In an untimely death case, the estimation might be very important for some legal matters such as inheritance and life insurance. Hitherto, only limited methods are applicable for estimation of the PMI. For the early PMI, algor mortis and supravital reaction are considered as the most applicable and reliable [1], [2], and livor mortis, rigor mortis and gastric content also are useful. For the late PMI, insect development is commonly considered as the primary and most accurate means for estimating the minimum PMI [3]–[5], despite of the potential of potassium and hypoxanthine in vitreous humor [6]–[8]. However, once the PMI exceeds the duration of pre-adult development of necrophagous flies with emergence of the adults, its determination is primarily based on duration of the pre-adult development and is very approximate [4]. Recently, some studies suggested that decomposition chemistry of cadaveric remains might be a promising method for determining the PMI [9]–[14].

Empty puparia (formed from the cuticle of the third instar larvae) of necrophagous flies, especially those of Calliphoridae species, are one of the most common insect remains in the advanced stage of cadaver decomposition. Empty puparia have been used only to reveal the season the death occurred, based on the seasonal activity of flies [15], [16], or to determine the minimum PMI based on duration of the pre-adult development. Previously, fly puparia were found to contain rich hydrocarbons [17], [18], whose composition showed significant time-dependent changes during the weathering process in the laboratory in *Chrysomya megacephala* (Fabricius) (Diptera: Calliphoridae) [19], suggesting great potential of fly puparial hydrocarbons for determining the PMI. Here we show the substantial and regular time-dependent changes in the puparial hydrocarbons during the weathering process in the field in *C*. *megacephala*.

## Materials and Methods

### Ethics Statement

The field experiment was permitted by SUMC. In the activity, only the fly puparia were released on the grassland. Because they are neither organism nor chemical materials, they did no harm to the environment.

### Insects

The puparia of *Chrysomya megacephala* (Fabricius) (Diptera: Calliphoridae) were obtained from a colony of insects originating from wild eggs collected in the city of Shantou, Guangdong, China in 2008. The larvae were fed with fresh pork in a plastic basin with the diameter of 35 cm and the height of 20 cm. The basin was kept in a larger plastic box containing a layer of dry sawdust to provide a dry environment for pupation of the flies. The puparia were collected daily after adult emergence and kept at −30°C until use.

### Weathering and Sample Collection

The puparia were placed on the grassland with sandy soil and some shrubs about 5 m high behind the campus of Shantou University in the city of Shantou, Guangdong, China in November of 2008. The puparia were then sampled every 5 d during the first 40 d, and then every 10 d until day 90. The samples were stored at −30°C until chemical analysis. During the total weathering process, the mean (range) of the ambient average daily temperature was 15.3°C (9.4–22.8°C), and the mean of the ambient average daily relative humidity was 68% (35–91%). In addition, the mean of the ambient average sunlight time was 5.58 h (0–10.00 h), and the total rainfall was 72.9 mm.

### Chemical Analysis

Forty puparia for each time point were cleaned with a small degreasant brush in distilled water, dried by suction onto a filter paper, divided into five equal groups (8 puparia in each group) and weighed. Cuticular hydrocarbons were extracted by immersing each group in a glass vial containing internal standards (n-C24 and n-C36, 200 ng respectively, Fluka) dissolved in 800 µl pesticide grade hexane (Dikma Technology Inc. USA) at room temperature for 10 min, with vortexing about 5 sec at the 1^st^, 5^th^ and 10^th^ min.

The extract was concentrated to dryness under a stream of nitrogen (99.999%) and dissolved in 50 µl (for puapria with 0–40 d of weathering) or 10 µl (for 50–90 d) hexane for chemical analysis. A chromatographic run using normal alkane standard (C_21_–C_40_, 4 ng/µl, Fluka) was performed daily to demonstrate the response of the normal alkanes and to estimate those of the other cuticular hydrocarbons.

Quantitative analysis of cuticular hydrocarbons was carried out using an Agilent 6820 gas chromatograph coupled to an FID detector with a DB-5ms capillary column (30 m×0.18 mm i.d., 0.18 µm film). Two µl of the sample was injected on the column using the splitless injection mode. Helium (99.999%) was used as the carrier gas with a column head pressure of 28.0 psi. The injector and detector temperatures were 280°C and 330°C respectively. The oven temperature was 100°C for 3 min, then to 230°C at 10°C/min, and then to 325°C (30 min) at 3°C/min.

An Agilent 7890A/5975C GC/MSD system was used to identify the chromatographic peaks with a DB-5ms capillary column (30 m×0.25 mm i.d., 0.25 µm film). Helium was used as the carrier gas with a column head pressure of 11.3 psi. Injector temperature was 280°C. The oven temperature was the same as above, except that the end temperature was 310°C. Mass spectra were obtained at 70 eV and the GC/MSD interface temperature was set at 310°C. The identification of cuticular hydrocarbons was based upon EI mass spectra and literature data [20]–[22].

### Data Analysis

For the normal alkane standard, a good linear relationship was found between response factor and equivalent chain length (ECL) at the range of n-C23 to n-C36 (*r* ^2^>0.99, *P*<0.05). Therefore, for the cuticular hydrocarbons, the response factors were considered to correlate linearly with the ECL, and were estimated using the internal standards. The mass of each peak in a sample was calculated, as well as the mass per gram of puparia. To stabilize the variance and achieve linearity of the regression function, ln-transformation was performed for further regression analysis after the addition of 1 to eliminate missing values from the CHC data set. The relative mass abundance (RA) of each peak to n-C27, n-C29, n-C31 and n-C33 was calculated: the geometric mean of 1.5×M_n-C27_ (mass abundance of n-C27, same for the below), M_n-C29_, 3×M_n-C31_, 6×M_n-C33_ was considered as the mass reference value (100), because the 4 peaks were relatively stable with a mass ratio of about 4∶6:2∶1 in the fresh puparia. As above, ln-transformation of the data was also performed after the addition of 1 to eliminate missing values from the CHC data set. Spearman correlation, linear regression analysis and lack-of-fit test were performed using SPSS for Windows 17.0. Results were considered as statistically significant if *P*<0.05.

## Results

**Figure 1:**
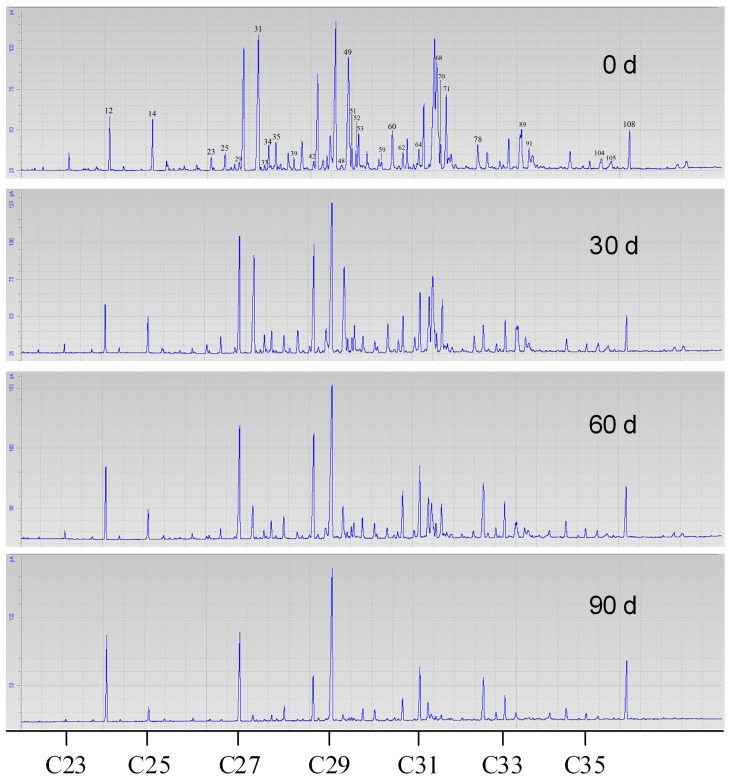
Changes in the chromatographic profile of the puparial hydrocarbons during the 90-day weathering process in *Chrysomya megacephala*. The puparial cuticular hydrocarbons were analyzed quantitatively using an Agilent 6820 gas chromatography coupled to an FID detector.

**Figure 2:**
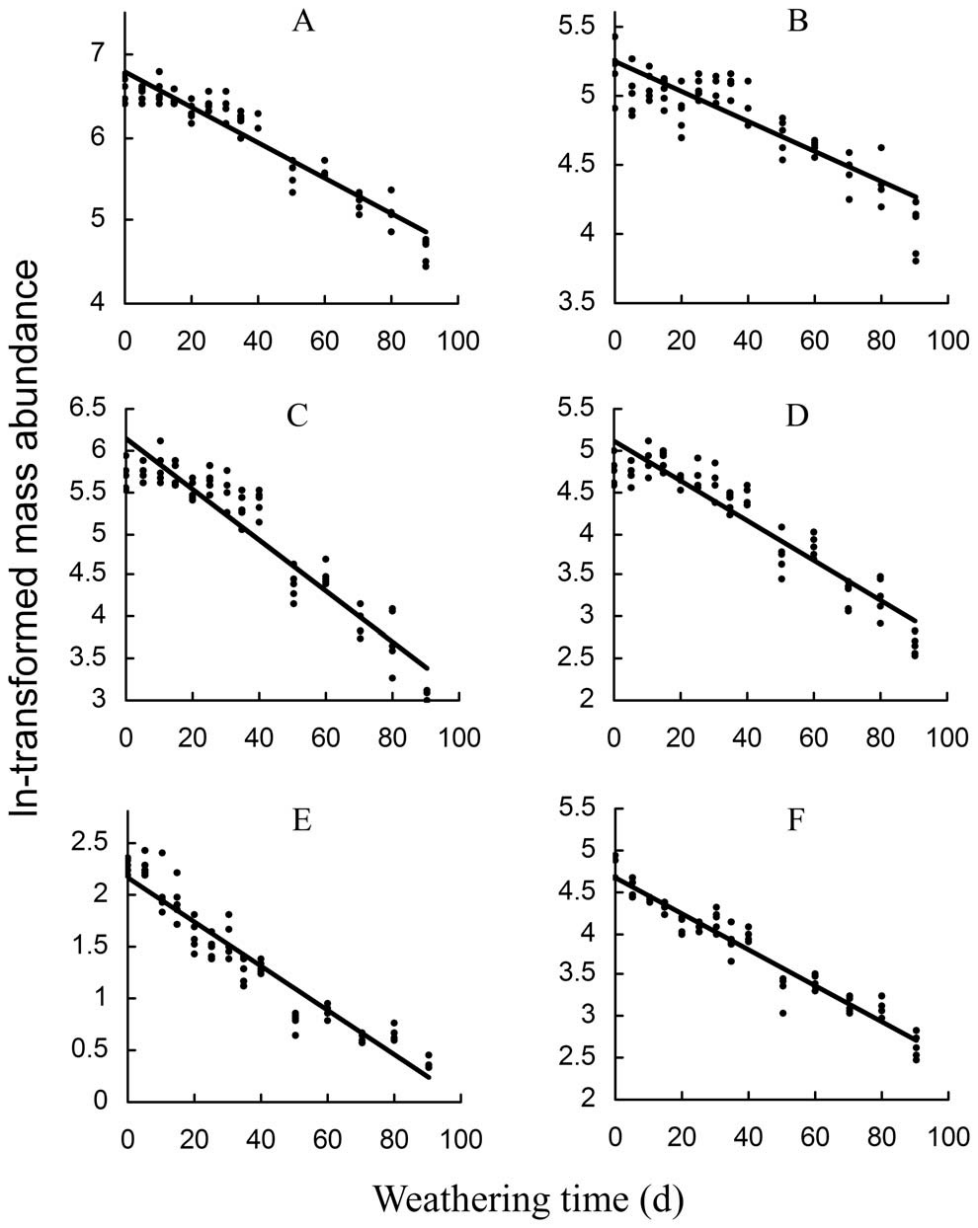
Time-dependent decreases in ln-transformed mass abundance of various classes of puparial hydrocarbons during the 90-day weathering process in *Chrysomya megacephala* (*P*<0.05). The corresponding functions were: A, total hydrocarbon, *y = *−0.0215*x+*6.8009, *r^2^ = *0.9019; B, normal alkane, *y = *−0.0107*x+*5.2353, *r^2^ = *0.7448; C, monomethyl alkane, *y = *−0.0306*x+*6.1457, *r^2^ = *0.8865; D, dimethyl alkane, *y = *−0.023*x+*4.9965, *r^2^ = *0.8814; E, Alkene, *y = *−0.0326*x+*3.2289, *r^2^ = 0.9063*; F, other unidentified lipids, *y = *−0.0217*x+*4.7088, *r^2^ = 0.9388*.

Hexane extract of the puparia was found to contain a mixture of n-alkanes, monomethyl alkanes, dimethyl alkanes, and alkenes with the carbon chain length of 23 to 35, as well as some other unidentified chemicals (Table 1) [19]. Significant changes were found in the chromatographic profile of the puparial hydrocarbons during the 90-day weathering process (Fig. 1). Of all the 106 hydrocarbon peaks (internal standards excluded), 104 decreased significantly with the weathering time in their abundance (Spearman correlation analysis, *P<*0.05; Table 1), except for Peak 80 (14, 18-; 12, 16-Dimethyl-C32) without significant change (*P*>0.05) and Peak 97 (Unidentified) with a significant increase. As a result of weathering, the total abundance of hydrocarbons decreased significantly to 14.12%, from 726.1±110.1 to 102.5±14.8 (µg/g puparium), and similar changes were also found in the various types of hydrocarbons. These relationships between ln-transformed mass abundance and weathering time were able to be modeled well with linear functions (*P*<0.05, Fig. 2).

**Table 1:** Composition of cuticular hydrocarbons of fresh puparia and their time-dependent changes during the 90-day weathering process in *Chrysomya Megacephala*.

Peak number	ECL	Hydrocarbon	Mass abundance (µg/g, Mean±SD)	*r* Value*	*P* Value*	Residual ratio (%)	*r^2^* ^#^	*P* Value#
1	23.00	n-C23	4.096±0.38	−0.9055	<0.001	8.37		
2	23.06	Unidentified	0.192±0.03	−0.8099	<0.001	0.00		
3	23.26	Unidentified	0.100±0.02	−0.7015	<0.001	6.56		
4	23.35	11-; 9-MeC23	0.592±0.04	−0.8167	<0.001	8.37		
5	23.39	7-C23	0.522±0.02	−0.9332	<0.001	0.00		
6	23.43	Unidentified	0.176±0.04	−0.7173	<0.001	5.56		
7	23.47	Unidentified	0.572±0.07	−0.9482	<0.001	3.90		
8	23.62	Unidentified	0.207±0.10	−0.6118	<0.001	3.84		
9	23.67	3-MeC23	1.025±0.16	−0.7049	<0.001	14.73		
10	23.77	Unidentified	0.424±0.03	−0.9175	<0.001	0.00		
11	23.92	Unidentified	0.158±0.02	−0.5599	<0.001	46.14		
12	24.00	n-C24						
13	24.05	Unidentified	0.359±0.02	−0.9668	<0.001	0.00	0.9012	<0.001
14	25.00	n-C25	13.72±2.60	−0.8563	<0.001	13.57	0.8332	<0.001
15	25.32	13-; 11-; 9-MeC25	3.805±0.50	−0.9518	<0.001	10.65		
16	25.39	7-MeC25	0.408±0.03	−0.9090	<0.001	0.00	0.8216	<0.001
17	25.48	5-MeC25+Unidentified	0.455±0.04	−0.9126	<0.001	11.33		
18	25.61	11,15-; 9,13-diMeC25	0.838±0.08	−0.9499	<0.001	1.20	0.8915	<0.001
19	25.72	3-MeC25	1.403±0.20	−0.9620	<0.001	2.50	0.9161	<0.001
20	25.78	Unidentified	0.396±0.07	−0.8501	<0.001	1.42		
21	26.00	n-C26	1.421±0.25	−0.8058	<0.001	23.95		
22	26.04	3,13-; 3,11-; 3,9-; 3,7-diMeC25	0.966±0.08	−0.9559	<0.001	0.53	0.8930	<0.001
23	26.31	13-; 12-; 11-; 10-MeC26	3.955±0.72	−0.9397	<0.001	1.42	0.8844	<0.001
24	26.36	6-MeC26	0.895±0.10	−0.7460	<0.001	33.32		
25	26.60	2-MeC26	4.352±0.85	−0.8666	<0.001	5.14	0.8312	<0.001
26	26.70	C27∶1	0.241±0.02	−0.7081	<0.001	0.00		
27	26.74	Unidentified	0.794±0.09	−0.9416	<0.001	0.84		
28	26.80	Unidentified	1.843±0.14	−0.9250	<0.001	0.09		
29	26.90	2,14-; 2,12-diMeC27	2.598±0.36	−0.9282	<0.001	1.30	0.8771	<0.001
30	27.00	n-C27	44.28±7.73	−0.7803	<0.001	27.26		
31	27.32	13-; 11-; 9-MeC27	52.12±10.26	−0.9279	<0.001	1.52	0.8793	<0.001
32	27.38	7-MeC27	0.69±0.09	−0.8656	<0.001	22.99		
33	27.47	5-MeC27	1.21±0.18	−0.9269	<0.001	1.64	0.8709	<0.001
34	27.56	11,15-diMeC27	6.45±1.11	−0.9156	<0.001	3.36	0.8613	<0.001
35	27.72	3-MeC27	7.43±1.00	−0.8753	<0.001	9.32	0.8512	<0.001
36	27.77	Unidentified	1.11±0.15	−0.9410	<0.001	0.50	0.8744	<0.001
37	28.00	n-C28	6.12±1.00	−0.8031	<0.001	27.13		
38	28.11	3,9-;3,7-diMeC27	3.49±0.32	−0.9521	<0.001	0.75		
39	28.28	14-, 13-, 12-, 11-, 10-MeC28	9.73±1.56	−0.9100	<0.001	1.96	0.8694	<0.001
40	28.35	8-MeC28	0.47±0.07	−0.3368	0.1817	1.83		
41	28.46	6-MeC28	0.10±0.01	−0.7763	<0.001	37.44		
42	28.53	4-MeC28	2.69±0.39	−0.8931	<0.001	4.67	0.8517	<0.001
43	28.62	2-MeC28	28.86±5.80	−0.7278	<0.001	20.98		
44	28.72	C29∶1a	4.29±0.26	−0.9628	<0.001	10.87		
45	28.80	C29∶1b	4.22±0.44	−0.9716	<0.001	0.23	0.8103	<0.001
46	28.87	2,14-; 2,12-; 2,10-diMeC29	11.95±2.15	−0.8929	<0.001	2.78	0.8407	<0.001
47	29.00	n-C29	70.97±14.41	−0.6887	<0.001	37.02		
48	29.12	Unidentified (CH)	2.23±0.36	−0.9148	<0.001	7.14	0.8089	<0.001
49	29.28	15-; 13-; 11-MeC29	46.88±7.71	−0.9122	<0.001	1.67	0.8176	<0.001
50	29.32	9-MeC29	3.95±0.58	−0.9388	<0.001	2.45	0.8976	<0.001
51	29.36	7-MeC29	4.60±0.71	−0.9173	<0.001	3.28	0.8477	<0.001
52	29.46	5-MeC29	4.53±0.38	−0.8777	<0.001	8.45	0.8407	<0.001
53	29.52	13, 17-diMeC29	10.68±1.64	−0.8986	<0.001	3.46	0.8456	<0.001
54	29.59	11, 15-diMeC29	1.28±0.22	−0.8702	<0.001	7.81	0.8101	<0.001
55	29.64	7,11-diMeC29	1.63±0.57	−0.8248	<0.001	4.16		
56	29.72	3-MeC29	5.58±0.83	−0.7873	<0.001	25.50		
57	29.75	5,9-diMeC29	1.39±0.25	−0.7140	<0.001	0.00		
58	30.00	n-C30	3.94±0.53	−0.7335	<0.001	36.66		
59	30.06	3,11-;3,9-;3,7-diMeC29	2.33±0.38	−0.8808	<0.001	5.13	0.8408	<0.001
60	30.29	15-; 14-; 13-; 12-; 11-; 10-Me C30	13.26±2.11	−0.9249	<0.001	2.48	0.8876	<0.001
61	30.39	6-MeC30	1.46±0.19	−0.7318	<0.001	33.10		
62	30.52	13, 17-; 12, 16-diMeC30	4.74±0.73	−0.9018	<0.001	4.66	0.8579	<0.001
63	30.62	2-MeC30	7.20±1.41	−0.4621	<0.001	39.38		
64	30.88	2,14-; 2,12-; 2,10-; 2,8-diMeC30 C30	6.44±1.24	−0.8726	<0.001	4.28	0.8228	<0.001
65	31.00	n-C31	21.30±4.33	−0.7094	<0.001	34.66		
66	31.12	Unidentified (CH)	1.63±0.32	−0.8189	<0.001	2.76		
67	31.24	Cholesterol	74.8±9.11	−0.9677	<0.001	5.27		
68	31.30	15-; 13-; 11-MeC31	50.55±10.14	−0.9014	<0.001	3.11	0.8750	<0.001
69	31.34	9-MeC31	5.73±1.05	−0.9156	<0.001	4.03	0.8878	<0.001
70	31.39	7-MeC31	6.25±1.05	−0.8756	<0.001	8.15	0.8604	<0.001
71	31.52	13,17-diMeC31	23.15±4.53	−0.9139	<0.001	4.12	0.8662	<0.001
72	31.58	11,x-diMeC31	3.13±0.65	−0.8343	<0.001	3.52		
73	31.64	7,11-diMeC31	5.58±0.93	−0.9310	<0.001	1.37	0.8832	<0.001
74	31.76	5,9-diMeC31	2.38±0.35	−0.7701	<0.001	15.49		
75	31.86	Unidentified (CH)	0.56±0.36	−0.7996	<0.001	4.65		
76	32.00	n-C32	1.51±0.24	−0.7797	<0.001	22.33		
77	32.06	3,11-; 3,9-; 3,7-diMeC31	0.63±0.12	−0.7176	<0.001	14.33		
78	32.28	16-; 14-; 12-; 10-MeC32	9.17±1.68	−0.9374	<0.001	2.72	0.9039	<0.001
79	32.35	8-MeC32	0.41±0.08	−0.8226	<0.001	4.16		
80	32.49	14, 18-; 12, 16-diMeC32	6.50±1.08	0.1888	0.1176	128.70		
81	32.61	Unidentified (CH)	1.18±0.25	−0.5415	<0.001	25.54		
82	32.79	Unidentified	2.82±0.54	−0.6783	<0.001	52.81		
83	32.87	Unidentified	1.45±0.33	−0.8818	<0.001	7.89	0.8286	<0.001
84	32.94	2,16-; 2,14-; 2,12-; 2,10-diMeC32	0.36±0.10	−0.7414	<0.001	1.38		
85	33.00	n-C33	10.29±1.63	−0.7754	<0.001	32.05		
86	33.11	Unidentified	0.93±0.33	−0.6454	<0.001	5.09		
87	33.16	Unidentified (CH)	1.23±0.17	−0.9132	<0.001	13.65		
88	33.27	Unidentified+17-; 15-; 13-MeC33	12.63±2.19	−0.9169	<0.001	12.24		
89	33.32	11-; 9-MeC33+Unidentified	11.75±2.39	−0.8848	<0.001	1.63	0.8520	<0.001
90	33.38	Unidentified	0.72±0.21	−0.9105	<0.001	8.95		
91	33.50	13,17-; 11,23-diMeC33	8.01±1.84	−0.8952	<0.001	5.26	0.8655	<0.001
92	33.58	x,y-diMeC33	3.20±0.71	−0.7193	<0.001	23.49		
93	33.68	Unidentified	1.81±0.23	−0.9555	<0.001	15.95		
94	33.79	Unidentified	1.66±0.35	−0.9219	<0.001	20.23		
95	33.85	Unidentified	1.60±0.37	−0.9054	<0.001	20.11		
96	34.00	n-C34	0.68±0.22	−0.8032	<0.001	40.05		
97	34.10	Unidentified	0.42±0.11	0.8344	<0.001	336.4		
98	34.28	16-; 14-; 12-; 10-MeC34	1.27±0.30	−0.8891	<0.001	14.39		
99	34.38	Unidentified	0.75±0.13	−0.6973	<0.001	22.77		
100	34.51	Unidentified	7.96±1.08	−0.9283	<0.001	25.20		
101	34.82	Unidentified	1.46±0.44	−0.8533	<0.001	18.06		
102	35.00	n-C35	2.55±0.53	−0.7579	<0.001	34.23		
103	35.10	Unidentified	0.45±0.33	−0.8902	<0.001	9.93		
104	35.29	17-; 15-; 13-; 11-MeC35	5.50±1.16	−0.8817	<0.001	11.67	0.8571	<0.001
105	35.54	x,y-diMeC35	5.73±1.15	−0.9233	<0.001	5.26	0.8108	<0.001
106	35.70	Unidentified	0.87±0.37	−0.7094	<0.001	36.80		
107	35.83	Unidentified	0.75±0.17	−0.4302	<0.001	0.00		
108	36.00	n-C36						

* Spearman correlation between ln-transformed mass abundance (µg/g puparium, with the addition of 1 to eliminate missing values from the CHC data set) and weathering time.

# coefficient of determination between ln-transformed relative abundance and weathering time. Geomean of 1.5×M n-C27 (mass abundance of n-C27, same for the below), M n-C29, 3×M n-C31 and 6×M n-C33 was used as the mass reference value (100), with the addition of 1 to eliminate missing values from the CHC data set.

**Figure 3:**
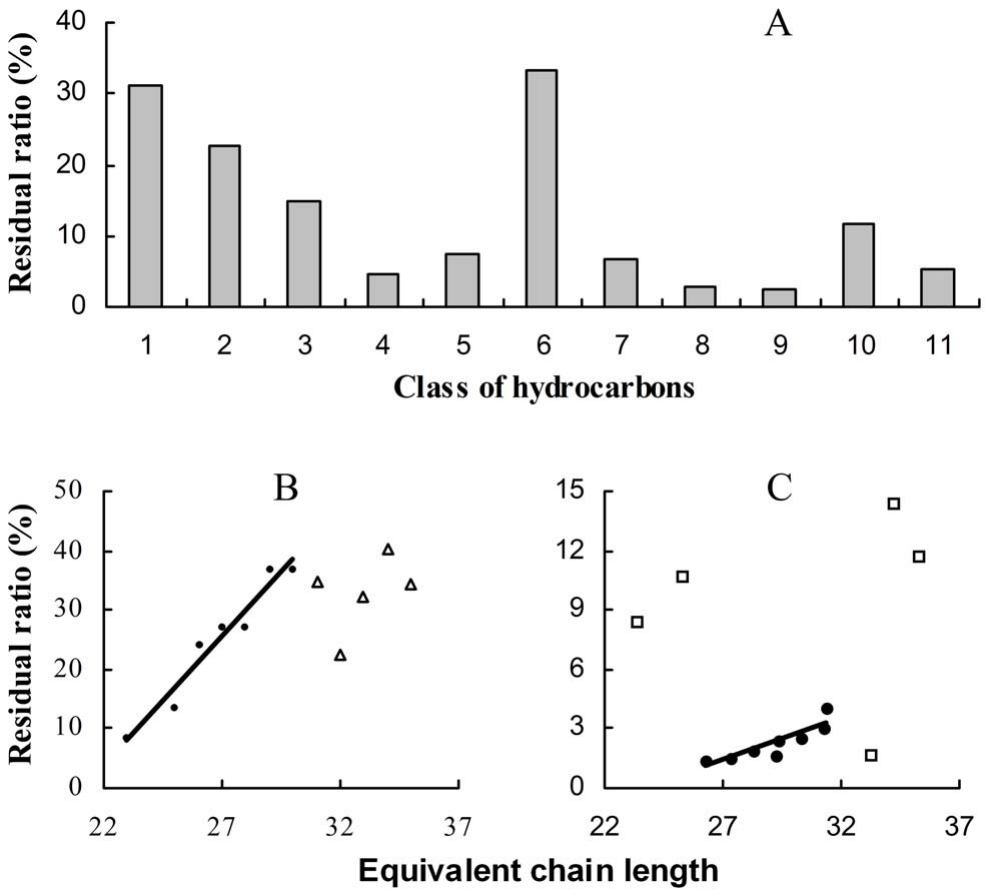
Effect of chemical structure and molecular weight (measured as equivalent chain length, ECL) on weathering rate of puparial hydrocarbons, here measured as the residual ratio (RR) within 90 d, in *Chrysomya megacephala*. **A**) RR of the hydrocarbons was found to depend on their chemical structure. 1, normal alkane; 2 to 8, monomethyl alkanes with methyl group at carbon 2 to 8; 9, internally branched alkane (with methyl group at carbon 9 or more); 10, dimethyl alkane; 11, alkene. **B, C**) RR of the hydrocarbons was positively correlated with equivalent chain length: **B,** normal alkanes; **C,** Internally branched monomethyl alkanes.

Weathering rate of the hydrocarbons, here measured as residual ratio (RR, negatively correlated with weathering rate) within 90 d, was found to depend on chemical structure. Normal alkanes seemed to be the most stable and had the highest RR (31.05%). For monomethyl alkanes (with general RR of 6.50%), RR had significant negative relationship with position of methyl group (Fig. 3A). The 2-methyl alkanes had the highest RR (22.55%), while the internally branched alkanes (with the methyl group on carbon 9 or more) had the lowest RR (2.64%). However, the 4-methyl and 6-methyl alkanes had unexpected RR of 4.67% and 33.35%, possibly due to their relatively low abundance and coelution with other components. The dimethyl alkanes and alkenes had relatively lower RR of 12.57% and 2.08%.

The RR of hydrocarbons was found to correlate with their molecular weight, here measured as equivalent chain length (ECL) (*r = *0.213, *P = *0.027). Firstly, RR of the normal alkanes increased linearly with ECL at the range of 23 to 30 (*r* ^2^ = 0.942, *P<*0.001), but thereafter had no obvious change (Fig. 3B). Secondly, RR of internally branched monomethyl alkanes had significant positive relationship with ECL at the range of 26 to 33 (*r* ^2^ = 0.6686, *P<*0.01; Fig. 3C). The unexpected RR beyond the range (Fig. 3C) may be due to the relatively low abundance and coelution with other components. The RR of the other hydrocarbons demonstrated a significant positive relationship with ECL (*P<*0.05).

**Figure 4:**
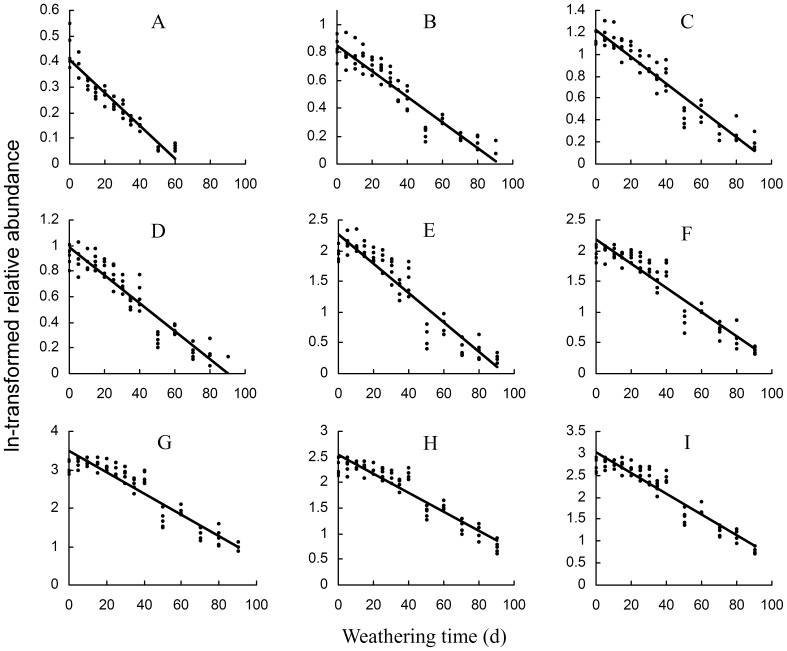
Time-dependent decrease of ln-transformed relative abundance of puparial hydrocarbons in *Chrysomya megacephala* during the 90-day weathering process, only for nine of those peaks whose variance in the abundance was explained best by weathering time. Geomean of 1.5×M_ n-C27_ (mass abundance of n-C27, same for the below), M_ n-C29_, 3×M_ n-C31_ and 6×M_ n-C33_ was used as the mass reference value (100). The corresponding functions were: **A**, unidentified (peak 13), *y = *−0.0064*x+*0.4043, *r^2^ = *0.9012; **B**, 11,15-; 9,13-DimeC25 (peak 18), *y* = −0.0092*x*+0.8518, *r^2^* = 0.8915; **C**, 3-meC25 (peak 19), *y* = −0.0122*x*+1.2223. **D**, 3,13-;3,11-;3,9-;3,7-DimeC25 (peak 22), *y = *−0.0109*x+*0.9816, *r^2^ = *0.893; **E**, 13-;12-;11-;10-meC26 (peak 23), *y = *−0.0241*x+*2.2721, *r^2^ = *0.8844; **F**, 9-meC29 (peak 50), *y = *−0.0198*x+*2.1825, *r^2^ = *0.8976; **G**, 15-;14-;13-;12-;11-;10-meC30 (peak 60), *y = *−0.0276*x+*3.4828, *r^2^ = *0.8876; **H**, 9-meC31 (peak 69), *y = *−0.0186*x+*2.5372, *r^2^ = *0.8878; **I**, 16-;14-;12-;10-meC32 (peak 78), *y = *−0.0237*x+*3.014, *r^2^ = *0.9039.

For the great difference in RR among the various hydrocarbons, their relative mass abundance (RA) (here to n-C27, n-C29, n-C31 and n-C33) was expected to be a better indicator for the weathering time and so was used in further analysis. The RA of nearly all the branched alkanes and alkenes decreased significantly over time (*P*<0.05, data not presented), but for two peaks (2-methyl-C30 and 14, 18-; 12, 16-dimethyl-C32). Additionally, for 40 of the 106 peaks, the ln-transformed RA decreased linearly with the weathering time with over 80% of the variance explained (Table 1, Fig. 4).

## Discussion

The puparial hydrocarbons of *Chrysomya megacephala* were found to weather significantly and regularly with time in the field. The weathering rate was found to depend on the chemical structure and molecular weight. The very regular changes support their application for determining the late PMI.

So far, for the later stages of decomposition, despite some recent studies on decomposition chemistry of cadaver remains [9]–[14], those currently available methods are not accurate enough. One of the primary reasons is the relative absence of field studies for these methods [2]. In fact, ethical issues preclude studies on human corpses in the late PMI. Therefore, fly puparia seem to be excellent materials to explore methods for determining the late PMI, because of its simultaneous occurrence with the adult emergence and its potential time-dependent changes. In addition, the composition of the cuticular hydrocarbons is theoretically predictable [23]–[25] in fresh puparia and changes regularly over time. This makes the puparial hydrocarbon method promising for determining the weathering time of the puparia and then for the late PMI. This method could prolong the determinable PMI from 2–4 weeks to several months.

In contrast to in the laboratory [19], the changes found here in the field were much greater during the weathering process of the puparial hydrocarbons in *C*. *megacephala*. The difference suggested that weathering of fly puparial hydrocarbons was influenced significantly by environmental factors, possibly including physical, chemical and biological ones, and biological factors may play a key role in the weathering. Without biological factors and other catalysts, branched alkanes will weather slower than n-alkanes for their relatively inactive chemical properties. Additionally, interaction of the various factors may also influence the weathering.

Except for the weathering process, the hydrocarbon composition of the weathered puparia are also determined by that before weathering, which might be influenced by various factors involved with the premature development, such as diet [26], temperature [27], humidity [28] or geographic origin [29]. However, hitherto no relative studies were reported in necrophagous flies.

Much further studies are required to demonstrate the effect of various factors on the composition of puparial hydrocarbons, before or during the weathering process, in different species of necrophagous flies. The effect of these factors is complex and difficult to deal with. In near future, it should be feasible to determine the weathering time of the puparia using the locally derived weathering data in the similar habitats and in the same seasons.

## Conclusions

We found cuticular hydrocarbon analysis to be a powerful tool for determining the weathering time of the puparia of necrophagous flies, and thus for estimation of the late PMI, despite various factors that might influence the composition of the puparial hydrocarbons, before or during the weathering.
